# Supplementary figures and images for: Cyclophosphamide Treatment Mimics Sub-Lethal Infections With Encephalitozoon intestinalis in Immunocompromised Individuals
Source: Front Microbiol. 2019 Sep 25;10:2205. doi: 10.3389/fmicb.2019.02205 (PMC6773878; doi:10.3389/fmicb.2019.02205)

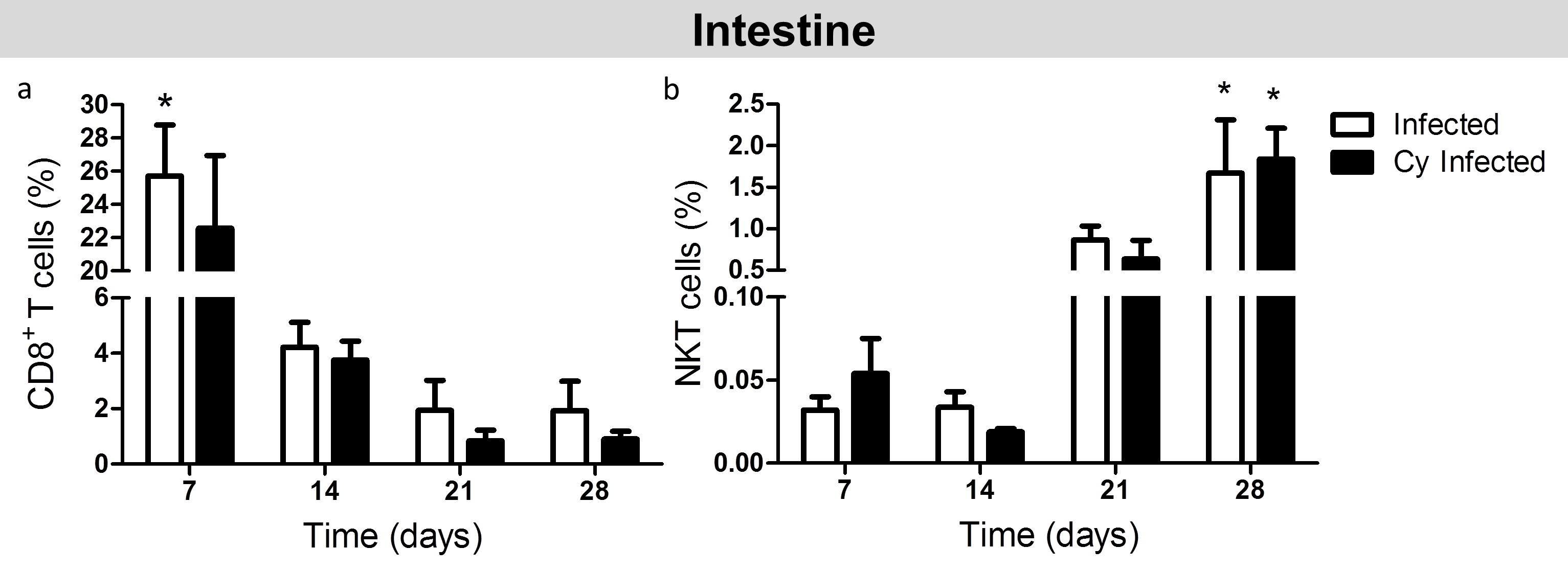

Supplement: FIGURE S1 — T cells population in the intestinal mucosa of mice infected with E. intestinalis and treated or not with Cy for 7, 14, 21, and 28 dpi. Percentage of lymphocytes (A) CD8 T (CD19– CD4–CD8+), and (B) NK/NKT cells (CD19– CD4+ NK1.1+). ANOVA test with Tukey’s post-test showed $p < 0.05, when compared to the same group at different dpi. [file Image_1.TIF]

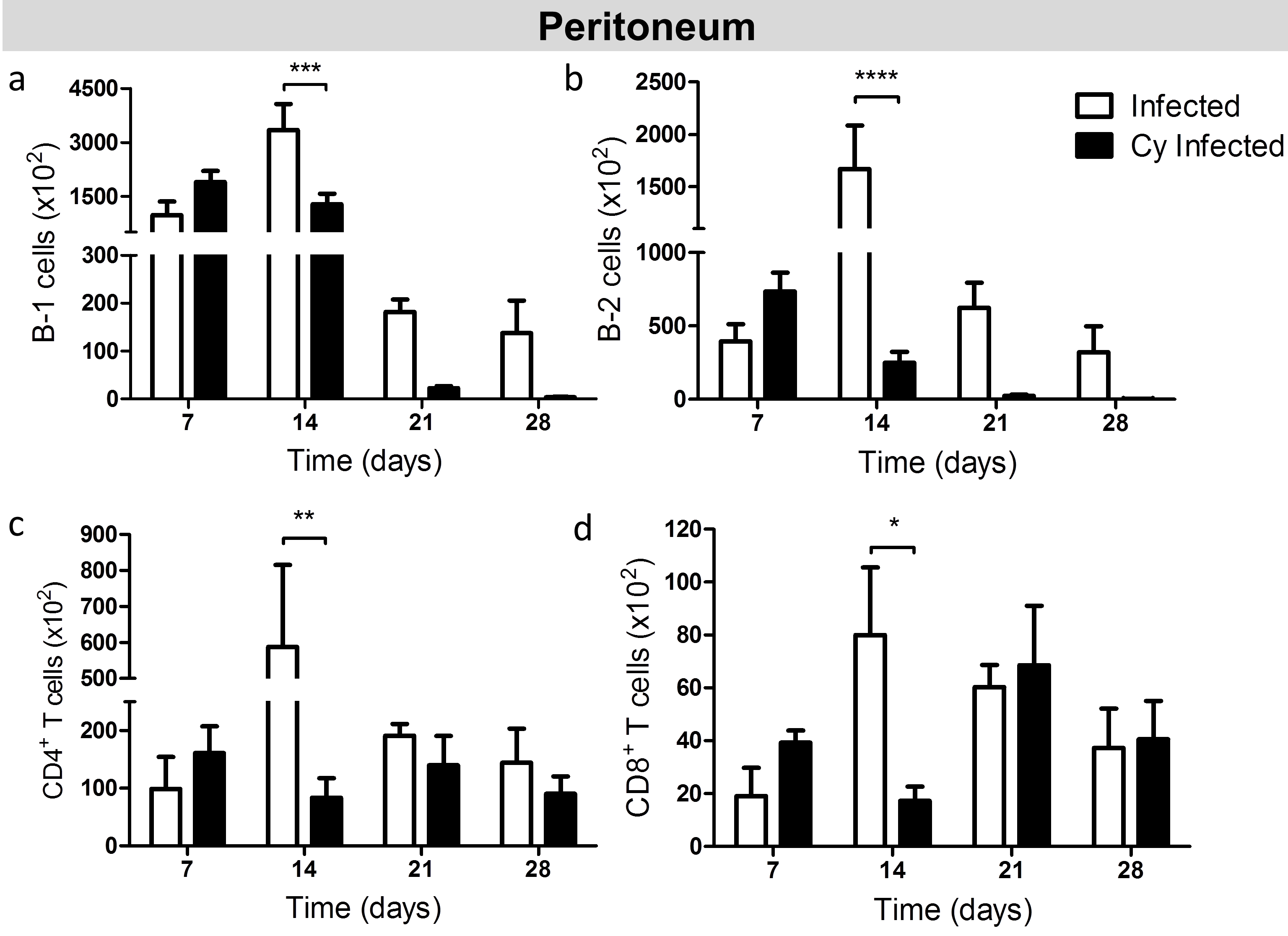

Supplement: FIGURE S2 — B and T cells into the peritoneal cavity of mice infected with E. intestinalis and treated or not with Cy for 7, 14, 21 and 28 dpi. (A) B-1 (CD23– CD19+), (B) B-2 (CD23+ CD19+), (C) T CD4 (CD19– CD8– CD4+), and (D) T CD8 (CD19– CD4– CD8+) cells numbers. ANOVA test with Tukey’s post-test showing ∗p < 0.05,∗∗p < 0.01, ∗∗∗p < 0.001, and ∗∗∗∗p < 0.0001. [file Image_2.TIF]
